# Efficacy of an absorbable polyglycolic acid patch in surgery for pneumothorax due to silicosis

**DOI:** 10.1186/1749-8090-7-18

**Published:** 2012-03-06

**Authors:** Xiao-Ming Lin, Yu Liu, Chuang Chi, Chao-Xi Lin, Yi Yang

**Affiliations:** 1 Department of Cardiac and Thoracic Surgery, the First Affiliated Hospital of Wenzhou Medical College, Wenzhou, China; 2 Department of Clinical Skills Center, Wenzhou Medical College, Wenzhou 325000, China

**Keywords:** Polyglycolic acid patch, Silicosis, Pneumothorax, Air leakage

## Abstract

**Background:**

We conducted a retrospective study to evaluate the efficacy and safety of an absorbable polyglycolic acid (PGA) patch in surgery for refractory pneumothorax due to silicosis.

**Methods:**

A retrospective analysis was performed of 56 patients who received thoracotomy or thoracoscopic surgery for refractory pneumothorax due to silicosis between 1995 and 2010. An absorbable PGA patch was used as a reinforcement or repair material after resection of the bulla in 24 operations and it was not used in another 32 operations. Clinical outcomes were compared between the two groups (with a PGA and without a PGA).

**Results:**

We found that the duration of postoperative chest drainage (5.04 ± 1.12 days vs. 8.19 ± 1.60 days, *p *< 0.01) and hospital stay after the operation (8.33 ± 1.34 days vs. 11.56 ± 1.50 days, *p *< 0.01) were significantly shorter in patients who used an absorbable PGA patch in the operation compared with those who did not use a PGA patch. The incidence of initial air leakage (58.3% [14/24] vs. 93.8% [30/32], *p *< 0.05) and relapse rate of pneumothorax in 6 months (4% [1/24] vs. 25% [8/32], *p *< 0.05) were also significantly lower in patients who used an absorbable PGA patch in the operation compared with those who did not use a PGA patch. No related adverse effects of the absorbable PGA patch occurred after the operations.

**Conclusions:**

Use of an absorbable PGA patch as a reinforcement or repair material in surgery for refractory pneumothorax due to silicosis can reduce postoperative air leakage and improve clinical outcome.

## Background

Quarrying is a prosperous activity in the mountainous area of Whenzhou, China. However, the local health conditions and occupational protection is poorer and the rate of silicosis is higher than those in other areas.

Pneumothorax caused by bulla rupture is a common complication of silicosis in stages II and III. Because of severe lung fibrosis, the rupture of bulla is rarely self-closed and pneumothorax usually requires surgical intervention. A typical operation involves resection of the bulla and suturing of the visceral pleura directly, but continual air leakage after the operation and a higher recurrence rate of pneumothorax are a large problem for thoracic surgeons.

Since 2005, we began to use an absorbable polyglycolic acid (PGA) patch as a reinforcement or repair material in surgery for refractory pneumothorax due to silicosis. Twenty-four patients received this procedure and the clinical outcome appeared satisfactory. The aim of this retrospective study was to evaluate the efficacy and safety of this material.

## Methods

During January 1^st^, 1995 to December 31^st^, 2010, 56 patients who received surgery for refractory pneumothorax due to silicosis at the First Affiliated Hospital of Wenzhou Medical College were recruited. All study data were collected retrospectively, and therefore, informed consent was not required.

The diagnosis of silicosis was based on case history and chest X-ray images. Twenty-one cases were in stage II silicosis and 35 cases were in stage III. The duration of chest drainage before the operation in all patients was more than 1 week. Fifty-six patients were divided into a study group, which used a PGA (Neoveil, Gunze Limited, Japan) in operations after 2005 and a control group, which did not use a PGA in operations before 2005.

**Figure 1:**
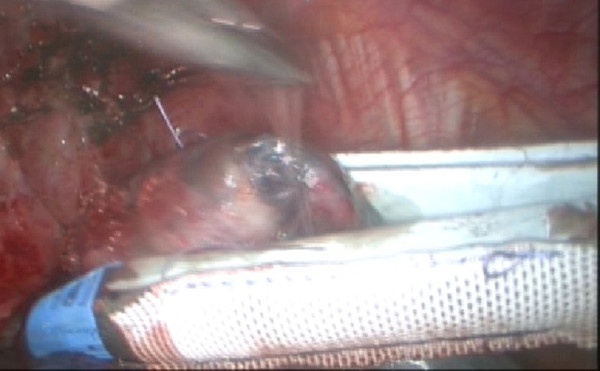
**Resected lung bullae with an endoscopic linear cutter, which buttressed with a sleeve-shaped PGA**.

**Figure 2:**
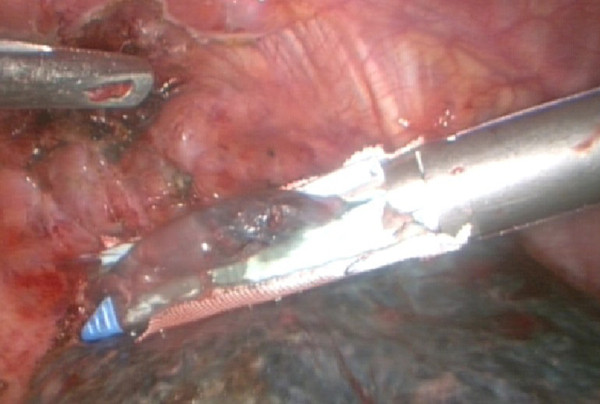
**Resected lung bullae with an endoscopic linear cutter, which buttressed with a sleeve-shaped PGA**.

**Figure 3:**
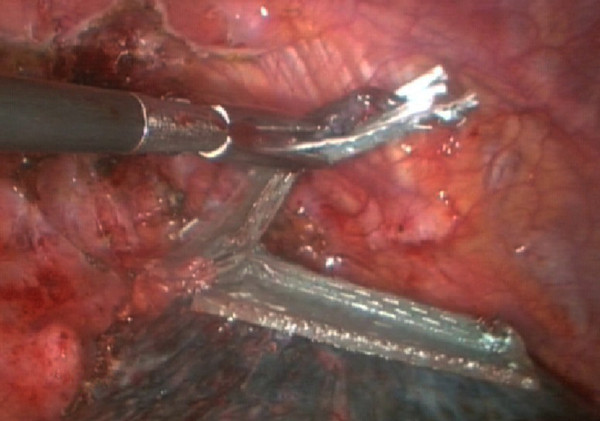
**Resected lung bullae with an endoscopic linear cutter, which buttressed with a sleeve-shaped PGA**.

**Figure 4:**
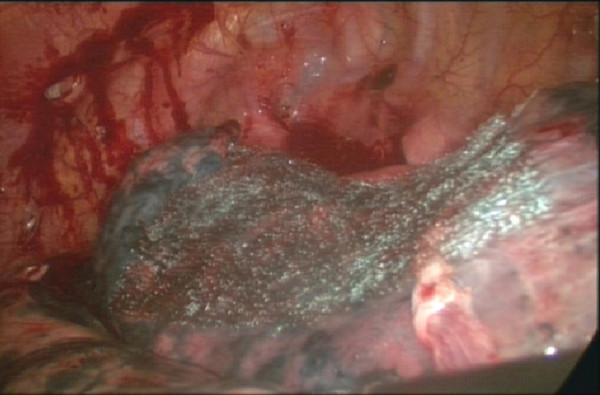
**The basilar part of the bullae was too wide after being resected, then used lamellar PGA and fibrin glue to repair the pleural defect**.

**Figure 5:**
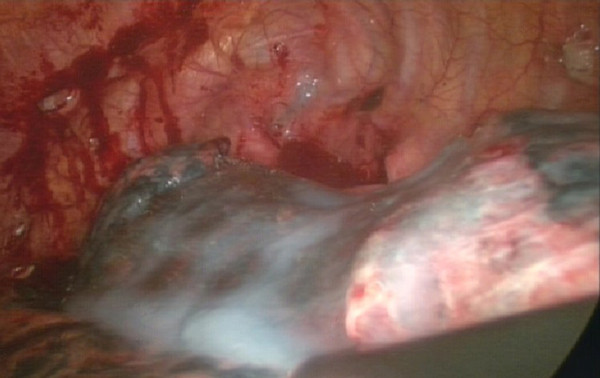
**The basilar part of the bullae was too wide after being resected, then used lamellar PGA and fibrin glue to repair the pleural defect**.

All surgeries were performed with general anesthesia and one-lung ventilation. In thoracotomy of the study group, after pleural adhesions were freed and lung bullae were resected, we sutured the visceral pleura continuously with a PGA strip as a buttress. If the pleural defect was too big to be sutured, we then used lamellar PGA to cover it and sprayed it with fibrin glue (RAAS Blood Products Co., Ltd, China). For thoracoscopic surgery of the study group, we resected lung bullae with an endoscopic linear cutter (Ethicon Endo-Surgery, USA), which buttressed with a sleeve-shaped PGA (Figure 1, Figure 2, Figure 3, Figure 4). If the basilar part of the bullae was too wide after being resected, we also used lamellar PGA and fibrin glue to repair the pleural defect (Figure 4, Figure 5). In the control group, all patients received thoracotomy, and after resection of the bullae, the visceral pleura was sutured with silk or prolene (Ethicon Endo-Surgery, USA) directly. Two intrathoracic drain tubes were placed in the operation. The incidence of initial air leakage, duration of postoperative chest drainage, hospital stay and the relapse rate of pneumothorax in 6 months in the two groups were compared, and we determined whether there were adverse effects of a PGA.

## Statistical analysis

Statistical analysis was performed using SPSS version 13.0. Data are presented as mean values ± standard deviation. Univariate analysis was conducted using the chi-square test for categorical data and the t-test for measurement data. Two-tailed p-values < 0.05 were considered statistically significant.

## Results

Perioperative data in all patients are presented in Table 1 and Table 2.

**Table 1:** Perioperative data in 56 patients

	PGA group (n = 24)	Control group (n = 32)
Age (years)	52.8 ± 10.4	56.6 ± 9.5
Male gender	24	32
Stage II (silicosis)	11	10
Stage III (silicosis)	13	22
Thoracotomy		
PGA buttress	12	0
Suture directly	0	32
PGA and fibrin glue	2	0
Thoracoscopic surgery		
Linear cutter buttressed with a sleeve-shaped PGA	8	0
PGA and fibrin glue	2	0

PGA: polyglycolic acid

**Table 2:** Comparison of outcomes between the two groups

	PGA group (n = 24)	Control group (n = 32)	*P*
incidence of air leakage	58.3%(14/24)	93.8% (30/32)	0.01
chest drainage(day)	5.04 ± 1.12	8.19 ± 1.60	0.00
hospital stay(day)	8.33 ± 1.34	11.56 ± 1.50	0.00
relapse rate	4%(1/24)	25%(8/32)	0.036

PGA: polyglycolic acid

The duration of postoperative chest drainage (*p *< 0.01) and hospital stay (*p *< 0.01) were shorter, and the incidence of initial air leakage (*p *< 0.05) and the relapse rate of pneumothorax in 6 months (*p *< 0.05) were lower in the study group compared with those in the control group.

## Discussion

Because of the combination of silicotic nodules and severe lung fibrosis, the texture of the lung in the advanced stage of silicosis becomes hard and Lack of flexibility. These characteristics induced by pneumothorax due to silicosis are not usually cured by chest drainage only and most patients need surgical intervention.

After surgical resection of lung bullae, suture of the visceral pleura is difficult. Excessive tissue tension induces tissue tearing by the sutures and persistent air leakage. This is considered to be the main reason of a long duration of postoperative chest drainage and recurrence of pneumothorax [1,2]. Therefore, use of proper reinforcement material should solve this problem.

Bovine pericardium has been widely used as reinforcement material in cardiothoracic surgery, and it can prevent air leakage [1,2]. However, it also has several obvious shortcomings. First, the preparation and preservation of bovine pericardium are too much trouble. Second, use of bovine pericardium as reinforcement material can cause severe pleural adhesions [1,2], and therefore, it's not suitable for a silicosis patient who has a high possibility of reoperation. After the mad cow disease in Europe, this material has not been able to be obtained in China. Because of its inability to degrade and cause pleural adhesions [1], we also consider that Teflon is not a suitable reinforcement material for use in surgery for pneumothorax due to silicosis.

Absorbable PGA is a type of polymer material, which has good biocompatibility. It can gradually degrade after absorbing water and can be absorbed completely by the human body 15 weeks after implantation. PGA is widely used in surgery as a type of absorbable repair and reinforcement material. It has different sizes and thickness, and is simple to apply. There are no reported increased risks of infection or pleural adhesions with PGA [3,4].

Our clinical outcomes showed that in surgery for pneumothorax due to silicosis, PGA can be used as reinforcement material and it reduces the tissue tension of sutures and minimizes tissue tearing. These effects can prevent air leakage, shorten the duration of postoperative chest drainage and reduce the recurrence rate of pneumothorax. If a tissue defect is too large to suture, a PGA patch and fibrin glue can be used to cover it directly. This avoids lung tissue damage by a forced suture and it prevents postoperative air leakage [4,5].

## Conclusions

In conclusion, we consider that an absorbable PGA patch is a very suitable reinforcement material for use in surgery for refractory pneumothorax due to silicosis, as it is both effective and safe.

## Abbreviation

PGA: Polyglycolic acid

## Competing interests

The authors declare that they have no competing interests.

## Authors' contributions

XML was responsible for the study design, data collection, statistical analysis, and drafting the manuscript. YL was responsible for data collection. CC was responsible for drafting the manuscript and revising it. YY was responsible for the study design and the final approval of the article. All authors read and approved the final manuscript.
